# Phenoliner: A New Field Phenotyping Platform for Grapevine Research

**DOI:** 10.3390/s17071625

**Published:** 2017-07-14

**Authors:** Anna Kicherer, Katja Herzog, Nele Bendel, Hans-Christian Klück, Andreas Backhaus, Markus Wieland, Johann Christian Rose, Lasse Klingbeil, Thomas Läbe, Christian Hohl, Willi Petry, Heiner Kuhlmann, Udo Seiffert, Reinhard Töpfer

**Affiliations:** 1 Julius Kühn-Institut, Federal Research Centre of Cultivated Plants, Institute for Grapevine Breeding Geilweilerhof, 76833 Siebeldingen, Germany; katja.herzog@julius-kuehn.de. (K.H.); nele.bendel@julius-kuehn.de (N.B.); reinhard.toepfer@julius-kuehn.de (R.T.); 2 Fraunhofer Institute for Factory Operation and Automation (IFF), Biosystems Engineering, Sandtorstr. 22, 39108 Magdeburg, Germany; hans-christian.klueck@iff.fraunhofer.de (H.-C.K.); andreas.backhaus@iff.fraunhofer.de (A.B.); udo.seiffert@iff.fraunhofer.de (U.S.); 3 Institute of Geodesy and Geoinformation, Department of Geodesy, University of Bonn, Nussallee 17, 53115 Bonn, Germany; wieland@igg.uni-bonn.de (M.W.); rose@igg.uni-bonn.de (J.C.R.); klingbeil@igg.uni-bonn.de (L.K.); heiner.kuhlmann@uni-bonn.de (H.K.); 4 Institute of Geodesy and Geoinformation, Department of Photogrammetry, University of Bonn, Nussallee 15, 53115 Bonn, Germany; laebe@ipb.uni-bonn.de; 5 ERO-Gerätebau GmbH, Simmerner Str. 20,55469 Niederkumbd, Germany; chohl@ERO-Weinbau.de (C.H.); wpetry@ERO-Weinbau.de (W.P.)

**Keywords:** big data, geo-information, plant phenotyping, grapevine breeding, *Vitis vinifera*

## Abstract

In grapevine research the acquisition of phenotypic data is largely restricted to the field due to its perennial nature and size. The methodologies used to assess morphological traits and phenology are mainly limited to visual scoring. Some measurements for biotic and abiotic stress, as well as for quality assessments, are done by invasive measures. The new evolving sensor technologies provide the opportunity to perform non-destructive evaluations of phenotypic traits using different field phenotyping platforms. One of the biggest technical challenges for field phenotyping of grapevines are the varying light conditions and the background. In the present study the Phenoliner is presented, which represents a novel type of a robust field phenotyping platform. The vehicle is based on a grape harvester following the concept of a moveable tunnel. The tunnel it is equipped with different sensor systems (RGB and NIR camera system, hyperspectral camera, RTK-GPS, orientation sensor) and an artificial broadband light source. It is independent from external light conditions and in combination with artificial background, the Phenoliner enables standardised acquisition of high-quality, geo-referenced sensor data.

## 1. Introduction

With new developments in electronics, software and sensor techniques, plant phenotyping has become a key technology in the agriculture sector. Platforms for the assessment of phenotypic data under controlled conditions are widespread [1,2,3,4]. These systems allow a very detailed assessment of plants under a controlled environment, genotype-environment interaction not taken into consideration. These systems are not however applicable for perennial crops, e.g., cultivated in trellis systems. Grapevine (*Vitis vinifera*), for example, is a large perennial liana that needs to be screened directly in the field for traits like plant architecture, yield, grape quality, abiotic and biotic stress. The application of non-invasive, sensor-to-plant methods facilitates the record of objective and repeatable phenotypic data of these traits. The lowest level of phenotyping applications are hand held devices applied by an operator. The Multiplex (Force A, Paris, France), a non-imaging fluorescence sensor was used to assess health status [5,6] or maturity [7,8] in grapevine. Some image-based hand-held prototypes have been used to evaluate yield parameters on grapes still attached to the vine using an artificial background and an RGB camera [9,10,11]. A completely different concept, being as distant as possible from the observed object, is the remote sensing approach using satellites [12], aircrafts [13], or unmanned aerial vehicles (UAVs) [14]. For most of these applications the focus is on plant health and water status, mostly expressed through vegetation indices, using mainly spectral sensors to validate the vigour of vineyards. In recent years, proximal sensing using moving vehicles as sensor carrier has been the biggest field of innovation in grapevine research. The simplest way is to attach sensors to tractors or smaller moving vehicles to monitor the crop for precision viticulture. This is for example done with commercial sensors like the GreenSeeker^®^ (NTECH Industries Inc., Ukiah, CA, USA) [15,16] or the Cropcircle (Netherlands Scientific Inc., Lincoln, NE, USA) [17], which are based on multispectral sensors, providing vegetation indices correlating with leaf area index (LAI) and canopy density. The GapeSense (Lincoln Ventures Ltd, Hamilton, New Zealand) uses digital images of the canopy side to assess height and texture [18]. Another opportunity is the use of LiDAR sensors for 3D reconstruction to validate the canopy size [19] and LAI [20]. Other more prototype-like approaches are cameras and light units mounted on movable vehicles like the approach by Nuske, et al. [21] for automatic yield estimations. The most automated approaches are the so-called “phenomobiles” [22] or “agbots” [23]. They use automation and robotic technologies to control the movement speed and direction within the vineyard, based on GPS or proximity sensors. They are either equipped with several sensors to monitor the plant status along the row and to gain phenotypic information or they can be used for agronomic operations. Approaches like the VineRobot [24], VINBOT [25] and the Wall-ye [26] are robots designed to in the end monitor various parameters non-destructively such as yield, vigour, water stress and grape quality using sensors ranging from RGB, multispectral, fluorescence to thermal and infrared. Most of them are still in the prototype stage, looking already very promising. The PHENObot [27] was recently introduced for the specific application in grapevine breeding to screen single vines in the grapevine repository for different phenotypic traits. Prototypes build for an agronomic concept are the VineGuard [28] which was designed for foliar applications and aims at implementing a robotic arm for harvesting grapes. Besides monitoring the vineyard Wall-ye [26] is designed to carry out precise pruning whereas the Vision Robotics Cooperation has developed an optical pruning system based on 3D reconstruction in a tractor pulled “tunnel” [29]. The Vitirover [30] is a small robot developed to automatically cut the grass within the row up to a 2–3 cm distance to the base of the vine. The usage of RTK GPS systems for geo-referencing is crucial for all field phenotyping approaches. Nevertheless, precision viticulture is focused on a whole plots level, whereas grapevine research requires a single vines resolution.

All of these phenotyping platforms have some challenges in common. The variation of the trait, for example berry colour, size and shape could differ, especially with regard to the evaluation of genetic resources in a repository. Therefore, the algorithms used for image analysis have to be very flexible [31]. Depending on the training system and the viticulture practice the occlusion through leaves or other grapes is challenging and needs to be considered when validating the sensor results. Screening vine rows in the side view is very challenging at the early phase of vegetation, as plants in images show the same colour distribution. Using a 3D reconstruction approach can help to overcome this point [32,33]. By far the biggest challenge in grapevine field phenotyping is the variable light condition. Some efforts have been made using artificial light units [21,27,28] to overcome the sun and doing the data acquisition at dawn or during the night [21,27]. Bourgeon, et al. [16] used an umbrella to avoid the Sun’s influence, whereas the Vision Robotics Cooperation [29] worked with a tractor pulled “chamber” to standardise the light conditions.

In the present study we developed a new, very robust field phenotyping platform, the Phenoliner, for applications in grapevine research and breeding. The biggest advantage of the Phenoliner compared to already used platforms is a standardized data acquisition, overcoming changing lighting conditions and changing background, by using a grape harvester as sensor carrier. We implemented two sensor systems, using RGB, NIR and hyperspectral imaging to classify different kinds of biological parameters optically and contact-free on the spot. Synchronic data acquisition and automated geo-referencing of high-resolute image data enable evaluation of several plant traits of whole breeding populations. Beside the Phenoliner setup, the accuracy of data acquisition and precision of geo-referencing was proved; the quality of sensor data was investigated on the example of bunch/berry detection and health status. 

## 2. Materials and Methods

For improved, sensor-based field phenotyping in vineyards the Phenoliner was developed. It consists of an emptied grape harvester as base, a differential GPS system and two sensor systems with their respective artificial light sources. The technical setup of the Phenoliner will be explained in the following paragraph.

### 2.1. Plant Material

Field tests were conducted in October 2016 in an experimental vineyard plot at the JKI Geilweilerhof located in Siebeldingen, Germany (49°21.747′ N, 8°04.678′ E). Rows were planted in north-south direction and consisted of 20 (*Vitis vinifera* cv. ‘Acolon’; experiment sensor A) and 24 (*Vitis vinifera* cv. ‘Riesling’; experiment sensor B) individuals. Inter-row distance was 2 m, and grapevine spacing was 1 m. At the southern end, the last four vines were not treated with chemical plant protection.

### 2.2. Vehicle

**Figure 1:**
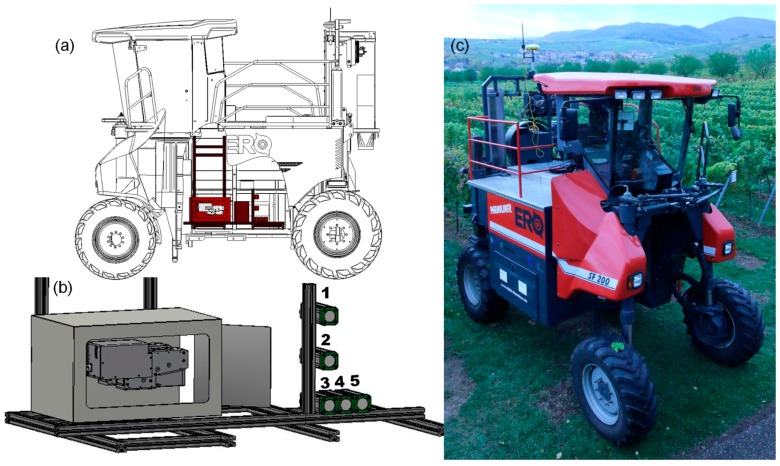
Overview of the Phenoliner. (**a**) Phenoliner construction plan. (**b**) Scheme of the sensor layout in the tunnel (marked red above); right: Sensor A: consisting of RGB cameras 1–3 and 5, and a NIR camera 4, left: Sensor B consisting of two hyperspectral cameras; (**c**) Phenoliner in the vine row.

The ERO-Grapeliner SF200 (ERO Gerätebau, Niderkumbd, Germany) was used as sensor carrier (Figure 1). All parts originally intended for the harvest, i.e., the shaking unit, destemmer, grape tank, and all parts used for the grape transport within the machine have been removed, including the hydraulic system needed for these parts. The emerging space was used for the sensor setup in the right part of the tunnel (minimal height: 2.05 m; maximum height: 2.80 m; width: 0.86 m). The space generated was used to integrate sensor systems and their peripheral components:

- In order to standardise the light conditions within the tunnel the base frame on the right hand side of the vehicle was extended and covered with metal plates. All slots in between were sealed and a curtain was installed in the back of the tunnel to avoid direct sun light interference
- Due to safety reasons on top of the machine the railing was enlarged where parts of the harvesting machine had been removed.
- The energy necessary for sensors, light units, and computer is provided by a generator driven by the vehicle. Two operating modes are possible: (1) diesel engine on and (2) diesel engine off. Due to the removal of the original harvesting hydraulics the free energy of the diesel engine can be used for powering a generator when the engine is on. Furthermore, it is possible to connect the vehicle to a regular power socket (230 V) when the engine is off. Two backup batteries (minimum 0.5 kWh) are bridging the time between turning off the engine and connecting the vehicle to the socket. This solution permits the transfer of the acquired data from the computers on the vehicle to the memory location without having the engine run for hours. There are 20 sockets available on the vehicle (cab: 2; front part: 9; back part: 9), provided with a suitable fuse through a distribution box.

### 2.3. RTK-GPS

All vines screened with the Phenoliner are surveyed using an real-time-kinematic (RTK)-GPS system (SPS852, Trimble^®^, Sunnyvale, CA, USA) with 2 cm accuracy. The geo-reference of each grapevine and the associated plant ID (unique for each plant) is stored in a database, Plant Location Administration (PLA) [27]. The GPS antenna is placed on top on the vehicle (see Figure 1c) and the receiver provides NMEA strings for both sensor systems: Sensor A: GGA 20 Hz; baud rate 115,200; Sensor B: GGA 20 Hz; GST 20 Hz; RMC 1 Hz; baud rate 38,400). The consideration of the lever arm between GPS antenna and the sensors is described below.

### 2.4. SensorA: Multicamerasystem (RGB, NIR)

Sensor A on the Phenoliner is a multi-camera system (MCS) consisting of four RGB cameras (DALSA Genie NanoC2590, Teledyne DALSA Inc., Waterloo, ON, Canada) and one near-infrared (NIR) camera (DALSA Genie Nano-M2590-NIR, Teledyne DALSA Inc., Waterloo, ON, Canada) arranged as shown in Figure 1b. Three of the RGB cameras (1–3, Figure 1a) are stacked vertically. Horizontally next to the lowest RGB camera 3, the NIR camera (4, Figure 1a) and afterwards the last RGB camera (5, Figure 1a) are positioned, with their protective cases touching each other. The cameras are equipped with 5.1 Megapixel sensors and 12 mm lenses. Given a distance of about 75 cm to the canopy, each camera covers an area of about 60 cm × 70 cm of the vine row, with a resolution of about 0.3 mm and a theoretical framerate of up to 51 frames per second. The illumination is realized using six 300 W halogen lamps (Hedler C12, Hedler Systemlicht, Runkel/Lahn, Germany), arranged around the camera system and pointing towards the canopy. In order to avoid hard shadows each lamp is equipped with a diffusor plate. All cameras are connected to a computer (Intel Core i7-860 with 2,8 GHz, 4 GB-DDR RAM, 2 × 480 GB SSD storage) via a GigE Interface. In order to enable the potentially high data rates, each camera is connected to a separate ethernet port and the images are stored on fast solid state disc (SSD) drives. For camera set up, camera control and synchrone image acquisition the IGG Geotagger 2.0 was developed in LabVIEW (National Instruments^®^ GmbH, Munich, Germany). It is a further development based on privious versions [27,34], and also provides precise georeference information for every single image using the GPS receiver and 2-axis inclinometer (DOG2 MEMS-Series USB Rev.1; TE Connectivity Sensors Germany GmbH, Dortmund).

### 2.5. Sensor B: Hyperspectral Camera System

Parallel to Sensor A that provides the three channels red, green and blue, the Phenoliner is equipped with Sensor B providing a total of 416 spectral bands covering a spectrum from 400 nm to 2500 nm. Sensor B consists of two separate commercially available line scanning hyperspectral cameras (Norsk Elektro Optikk AS, Skedsmokorset, Norway) covering the visual-near infrared range (HySpex VNIR 1600) from 400 to 1000 nm providing 160 channels across a continuous visible part of light and the short-wave infrared range from 1000–2500 nm (HySpex SWIR 320m-e, Norsk Elektro Optikk AS, Skedsmokorset, Norway) equally distributed over 256 channels. With an achievable frame rate of 160 Hz (VIS) and 100 Hz (SWIR) the combination of both cameras and the high spectral sampling rate of 3.2 nm and 5.45 nm, respectively, allows for a continuous acquisition of 16 Bit digitized high resolution reflectance data. The available space within the tunnel is limited, resulting in a maximum distance of 1m between lens and the vine canopy. Therefore both cameras are equipped with lenses of 1 m fixed focal length. To match the focal length, the hyperspectral line cameras are setup alongside the driving direction with a rectangular mirror diverting the reflected light to a 90° angle (Figure 1b). The optical industry provides silver- and gold-coated VNIR- and SWIR-specific mirrors adjusted to a highly constant reflectance of >95% across their entire respective spectrum but also low-cost mirrors have proven suitable for the task. Additionally, an artificial illumination of two 300 W short-wave spotlights (Hedler C12, Hedler Systemlicht) with a broad power spectral density were installed. In order to measure reflectance a 1 × 1 m spectralon with certified reflectance values (Sphere Optics, Hersching, Germany) was set up in the background (left tunnel side). It is covered with a custom foil specifically designed for the purpose of reducing absorption and retaining spectral features as much as possible while at the same time providing suitable mechanical protection for the pad. A previously conducted spectral measurement of the foil-covered pad gave satisfactory results.

## 3. Application of the Phenoliner: Pilot Study

### 3.1. Sensor A

#### 3.1.1. IGG Geotagger 2.0: Geo-Referencing of Images

The software enables accurate geotagging of all acquired images and the selection of images of single vines, when their coordinates are provided by the database (PLA). This is important with regard to automated data management. The coordinates of the vine stem from the database and the known offset direction between the stem and the area of interest (e.g., middle of the cane, bunch zone). This selection procedure enables significant reduction of storage space in the cases where not all images are needed. It also allows a direct association between the images and the database records of the grapevines. 

**Figure 2:**
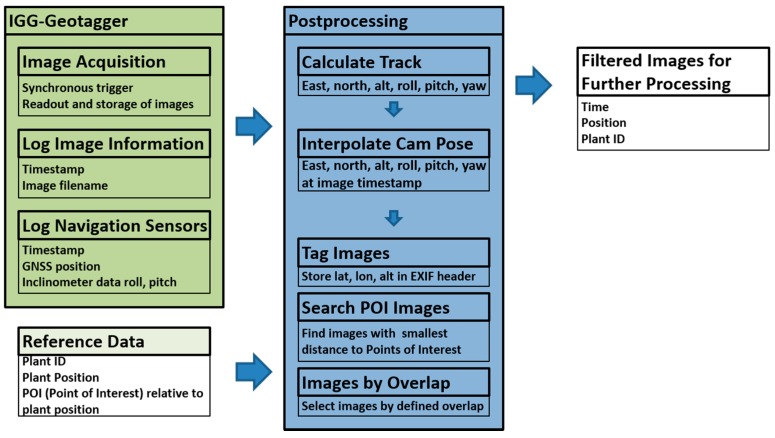
Tasks within IGG (Institute of Geodesy and Geoinformation) Geotagger 2.0. GNSS: Global Navigation Satellite System; Plant ID: Plant identification; EXIF: Exchangeable image file format.

There are several processes running on the image acquisition system and within the geotagging software (see Figure 2): During the motion of the vehicle through the vine row, the camera system is acquiring time synchronized images from every camera with a preconfigured frame rate and storing them to the SSD. If all cameras are used, the frame rate is limited to about 5 Hz, mainly due to the SSD writing speed. At the same time GPS positions (20 Hz) and inclinometer readings (roll and pitch angles, 20 Hz) are stored. 

In a post processing step, a full 6D (position and orientation) trajectory of the system is calculated from these data. The missing third rotation angle (heading) is estimated based on the sequence of positions and the assumption, that the Phenoliners motion direction is restricted to its long axis. This trajectory is then interpolated to the time steps of image acquisition. Knowing the lever arm between the GPS antenna and the cameras and its orientation now allows the calculation of a coordinate for each image, which is then written into the metadata of the image file. It should be noted here, that the determination of the roll and pitch angles of the system are a crucial step, because the angles can be controlled by the driver while driving through the rows. This means, that the angle between the camera system and the ground cannot be assumed to be small and constant as it may be possible for other ground vehicles.

In a further preprocessing step an image filter can be applied to reduce the number of images based on the purpose of the application. For the 3D reconstruction of the full vine row (see below) a selection based on minimum overlap between neighbouring images can be applied. To select images of a certain point of interest (POI), such as the bunch zone of single vines, a distance between the coordinates of the camera image center and the POI is calculated. For every POI the image with the minimal distance is selected.

#### 3.1.2. Validation

**Figure 3:**
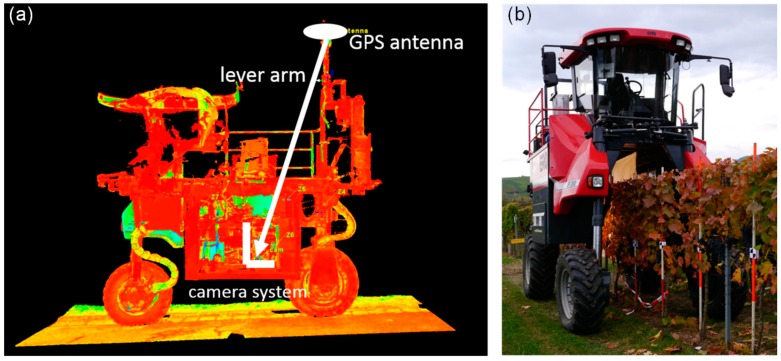
(**a**) Terrestrial laser scan of the Phenoliner to measure the lever arm between the GPS antenna and the camera system. (**b**) Evaluation measurement to test the georeferencing accuracy of the system. The coordinates of the black and white targets at the poles are known and can be compared with the target positions seen in the images.

As mentioned above, the lever arm between the GPS antenna and the camera system has to be known precisely, in order to calculate the image coordinates from the position and orientation measurements. This lever arm has been measured with an accuracy of millimetres using a 3D terrestrial laser scanner (Leica P20; Leica Microsystems GmbH, Wetzlar, Germany). Figure 3 shows the scan of the Phenoliner used for the lever arm determination.

**Figure 4:**
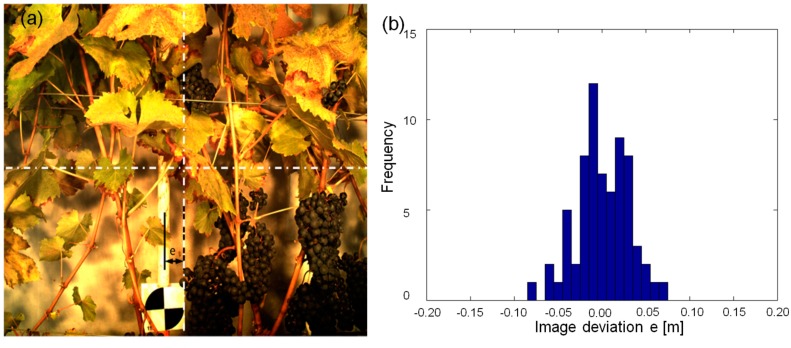
Experiment set up of POIs at the position of vine stems. (**a**) Image, selected as the one closest to an POI, with the deviation e as the error of the selection process. (**b**) Distribution of deviations from 10 runs at different speeds.

The experiment is driven by the need to automatically take images of a certain POIs, such as the bunch zones of single vines, having this POI in the middle of the field of view of the camera. Within one vine row (*Vitis vinifera* cv. ‘Acolon’, 20 vines), black and white targets were attached to poles at the position of the vine stem to mark a POI (see Figure 4a). The exact position of these poles was surveyed using an RTK GPS receiver and their coordinates were given to the software as “Reference Data” (Figure 2). Then the vehicle was driving through the row, taking five images per second. The geo-reference of each image was determined by calculating the trajectory and using the determined lever arm and the tagger software selected one image for every POI as described above. Figure 4a shows one of these images. Here the distance of the target (POI) to the vertical central axis of the image is considered as the “deviation” of the measurement. Please note, that we only evaluate the accuracy of one dimension, which is the one in the driving direction. The other two dimension are not relevant in this particular application, since the vertical field of view of the camera system is big enough to cover the whole canopy and the distance to the canopy is more or less constant due to the given row geometry. There is also no reason to assume, that the other two dimensions are less accurate than the evaluated one, as the accuracy in driving direction is the most critical due to time synchronization effects and the limited framerate (see below). To ensure the functionality of the system calibration and the image georeferencing procedure, an evaluation measurement was conducted.

This experiment has been performed five times at two different vehicle speeds (0.2 m/s and 0.4 m/s) each of them driving in both directions of the row. The distribution of the deviations for the 20 POIs in all runs is shown in Figure 4b. The overall accuracy of this POI based selection process is shown to be in the order of a few centimetres. However, note that this accuracy contains the accuracy of the image tagging procedure (trajectory calculation, calibration), the accuracy of the target position determination (RTK GPS) and the minimum distance between two consecutive images (vehicle speed, frame rate). The two different speeds combined with the frame rate of 5 Hz lead to an image distance of 4 cm and 8 cm limiting the image selection resolution to 2 cm and 4 cm, respectively. Given that the maximum deviation in these experiments is about 6 cm, we can assume the accuracy of the image tagging process itself is in the order of 2–3 cm, which corresponds to the expected accuracy of the GPS receiver.

#### 3.1.3. Application Example: 3D Reconstruction of the Full Vine Row

As shown in Figure 1b RGB cameras 1–3 are arranged vertically. These are used for a full 3D reconstruction of the vine row using multi-view stereo approaches [35]. These methods enable the reconstruction of 3D point clouds based on multiple overlapping images. This overlap is about 70–80% in both, the horizontal and vertical directions of the images. The vertical overlap is realized by the arrangement of the cameras. The horizontal overlap is realized by the motion of the Phenoliner. Here the combination of driving speed and image frame rate has to ensure a maximum distance of about 15 cm in order to achieve the required overlap. A vehicle speed of 0.2–0.3 m/s and an image frame rate of 5 Hz has shown to be a suitable parameter setup in practice.

**Figure 5:**
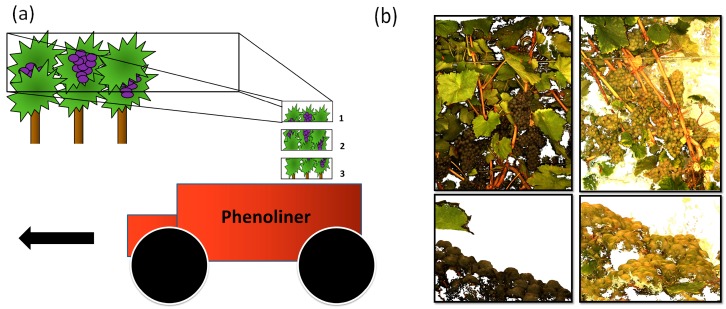
(**a**) Basic principle of the system setup for 3D reconstruction of the full vine row. The Phenoliners tunnel is driven over the vine rows. The MCS is oriented parallel to the rows, capturing images automatically while in motion. (**b**) Reconstructed point cloud of black (left side) and green grape (right side) varieties. The upper images show the rows from a frontal view and the lower images show grapes from a profile view. Single berry elevations of their spherical geometry are clearly distinguishable.

Abraham, et al. [36] captured images with the PHENObot [27] and used them for three-dimensional (3D) reconstruction of vine rows. In this study images were accordingly acquired using the Phenoliner to test transferability of developed workflow. Figure 5 shows the principle of the image acquisition for point cloud reconstruction using the vertical MCS (a) and black and green grapes (b) from the front (upper images) and in profile (lower images), that have been reconstructed from the images using the Software Pix4D. The black grape varieties have been gathered deploying a heavy grey tarpaulin as background while the green grape varieties have been gathered deploying a white heavy blanket as background. The choice of the background color has a significant impact on the quality of the 3D-reconstruction and is discussed in Section 3.1.3. The geo-reference of the images has been incorporated in the processing procedure, so each point in the point cloud has also a metric coordinate, enabling measurements with correct scale within the data.

As described in Abraham, et al. [36] these point clouds now undergo further processing steps, where the data are segmented and classified in order to provide information about the number of grapes and berries. Individual berry elevations are clearly visible, highlighting the high level of geometric detail of the point cloud. This level of geometric detail is necessary to count single berries via geometric modelling.

#### 3.1.4. Application Example: Depth Map Creation and Segmentation of Single Vines

Another application is the acquisition of a single image or stereo image pairs of single vines. While the application for the point cloud reconstruction needed images of the whole row with a sufficient overlap in horizontal and vertical direction, this application needs only selected images of a certain area of interest, for example the bunch zone. Calibrated cameras 3–5 are arranged based on the PHENObot experiences [27]. Camera calibration was performed by using a test field calibration according to [35] in order to determine the camera constant, principle point and camera lens distortions. No approximate values are necessary for this process. Afterwards, the internal parameters of the cameras are known enabling post processed image rectification. The rectified images then strictly follow the pinhole camera model with principle point in the centre, thus enabling increased precision for the subsequent steps. 

**Figure 6:**
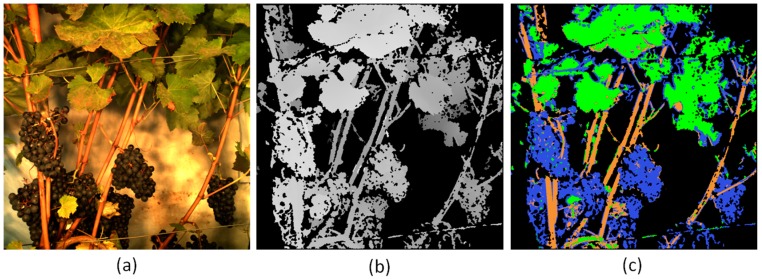
(**a**) Rectified image acquired with Sensor A (camera (5), only overlapping part of stereo pair shown). (**b**) Depth map calculated with stereo pair from camera (3) and camera (5). The brightness indicates the distance to the cameras (white for near points, dark gray for far points). Black pixels indicate positions with no depth which can be assumed to be background. (**c**) First result of a test for classification with manually set thresholds. The RGB image from (**a**) and the depth map from (**b**) are used as input. Classes are: blue for “grapes”, green for “canopy”, brown for “cane”, black for “background”.

The RGB image tools to detect berry size and colour as shown by [27,37] are currently adapted to Phenoliner images. First experiments regarding the computation of depth maps and segmentation of images, using stereo images acquired with the Phenoliner are showing promising result (see Figure 6). For these first tests the depth map has been calculated with the free software “pmvs2” [38] and is used to seperate foreground and background. Manually set thresholds for the colour channels of the RGB image are used to discriminate the other classes (“cane”, “canopy”, “grapes”). The artifical lightning may help to use a classifier with constant parameters over several data sets, because the brightness, constrast and colour temperature of the images will not change. Adjusting the published tools to the new sensors, the stereo system (cameras 3 and 5) can be used to segment an RGB image into the classes “cane”, “canopy” and “background” in order to calculate phenotypic parameters in the same manner as by Kicherer, et al. [32] for pruning mass or Klodt, et al. [33] for leaf area. For a detailed explanation of the stereo system layout of cameras 3 and 5, please refer to Kicherer, et al. [32]. The NIR-camera 4 is meant to be used for plant disease detection purposes and to improve the colour segmentation of different classes like canopy, cane, and grape bunches.

### 3.2. Sensor B

#### 3.2.1. Hyperspectral Image Acquisition

Image recording was achieved using proprietary image acquisition software implemented by Fraunhofer Institute for Factory Operation and Automation IFF, which integrates both hyperspectral cameras and the Phenoliners GPS receiver in order to record georeferenced hyperspectral images. 

**Figure 7:**
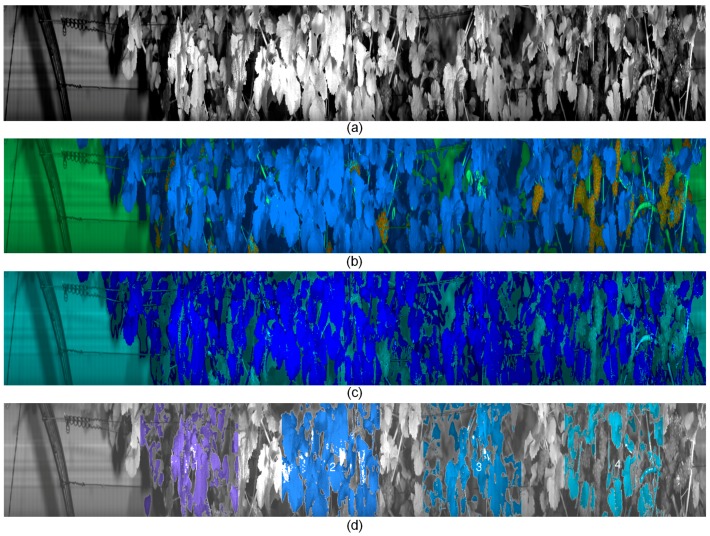
Pre-processing of hyperspectral image recording: (**a**) channel image at 1100nm, (**b**) clustering of spectral data, colour indicates pixel groups, (**c**) classification for foreground (leaves) vs. background, (**d**) image after removal of dark areas and marking of vine position from GPS.

Data pre-processing and analysis was done offline using Matlab 2013a (The MathWorks, Natick, MA, USA). Spectral data per image was clustered using a Neural Gas algorithm [39]. Spectra are grouped due to their similarity measured by the Euclidean distance to a number of prototype spectra, which are optimized to achieve minimal quantization error. The cluster or group that is representing plant material is selected and the segmentation mask is further processed using morphological eroding operations. Finally, a classifier Artificial Neural Network is trained on examples of leave spectra and background spectra. This classifier is applied to all images and achieves an automated classification of plant materials in all images. Region of interests in the image representing single vines are marked using the geo information recorded along the camera system. In order to obtain the dataset for the subsequent machine learning, a list of vines with the status “sprayed” and “not sprayed” was provided. Among all vines representing one of the two classes, 10,000 spectral pixels were sampled. Datasets are treated separately for the VNIR (160 features) and SWIR (256 features) camera. No pixel averaging was performed. In Figure 7 examples of the principal pre-processing steps are depicted.

The image segmentation can be performed in real-time on the vehicles computer system and will be the first processing component for the Phenoliners in-field detection capability of plant diseases based on leave spectral reflectance pattern. Since we image a geometrical complex scene, leaves can be overexposed or shadowed by other leaves. In order to decrease the variance in the spectral signal and to reduce noise, dark areas are segmented out (Figure 7d).

#### 3.2.2. Validation

**Figure 8:**
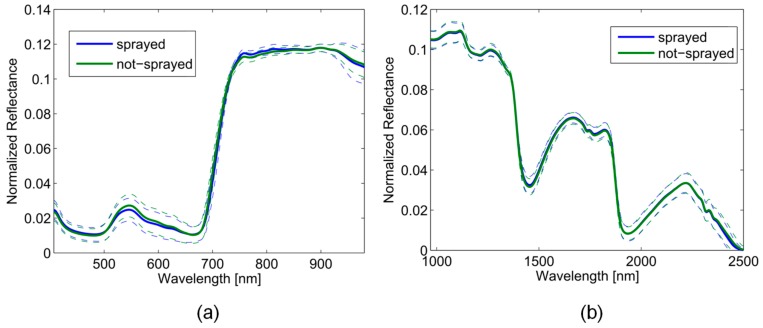
Normalized reflectance spectra for the VNIR 400–1000 nm (**a**) and SWIR 1000–2500 nm (**b**) range imaged at 2 p.m. and pooled over west and east side.

Field tests were conducted in October 2016 in one row of 24 individuals of *Vitis vinifera* cv. ‘Riesling’. Since the cameras were located on the right side of the movable tunnel, the row was recorded from the right (in the direction of motion). The zone around the grapes was scanned from the west and east side, respectively. Scanning was performed at 0.1 m/s. Data was acquisitioned at different time points during the day: 10 a.m., 12 p.m., 2 p.m., 4 p.m., and 6 p.m. In Figure 8 the averaged normalized reflectance pattern for both spray classes, measured from east and west at 2 p.m. are depicted, (a) in the range of 400–1000 nm and (b) 1000–2500 nm.

The final dataset of labelled spectral data per time point is then analyzed for discrimination of spray status (last four vines have not been sprayed) via a Linear Discriminant Analysis (LDA). An LDA finds a data projection that maximizes the class discrimination by maximizing between class distances and minimizing within class variance. Beyond a simple linear discrimination, a number of machine learning models were tested. On a dataset of labelled spectra, a Partially Least Square (PLS) model [40], a Radial Basis Function (RBF) network [41], a Multi-Layer Perceptron with linear output (MLP) [42] as well as soft-max output layer (PNET) [43] were performed (Table 1).

In order to evaluate generalization performance of the used machine learning algorithms, a five-fold cross validation was performed. For this purpose the data set is divided into five parts of equal sample size. Additionally the number of samples for both spray classes per fold is equalized. A model is then trained on four folds and tested on the fifth fold. All possible combinations are run and result in a mean accuracy for the test data over all folds. Mean accuracy and standard deviation are then used to determine the best machine-learning model. In Table 1, achieved prediction accuracy on the test folds are shown. A comparison of different machine learning approaches is worthwhile since as clearly indicated by the results, method can differ greatly in performance. 

**Figure 9:**
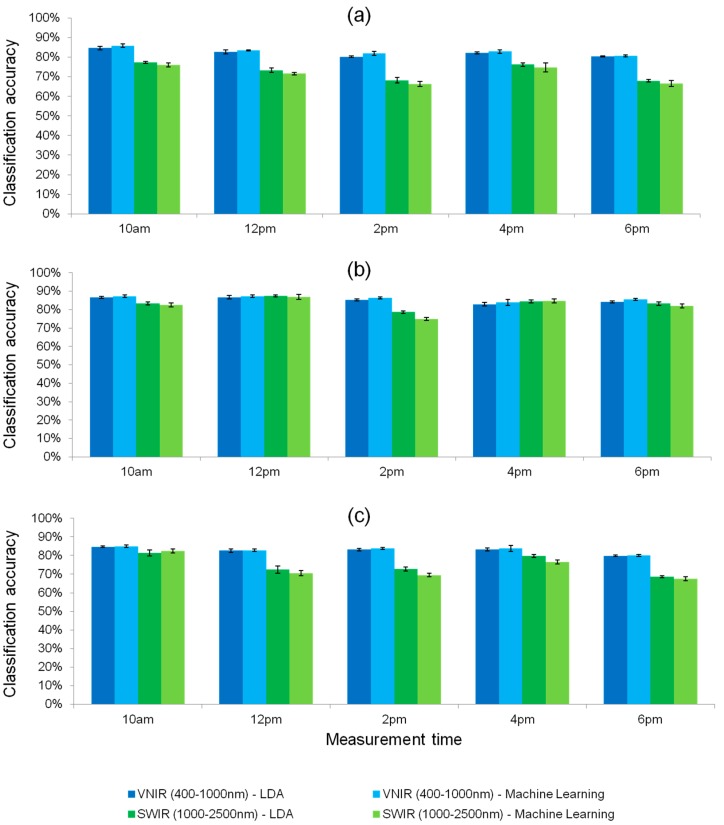
Classification accuracy for differentiation of sprayed vs. non-sprayed leaves measured from (**a**) west and east; (**b**) only west and (**c**) only east side. Across the results, the spectral reflectance data of the VIS-NIR range seems to be the more robust predictor for the spray status. Machine Learning and LDA approach are compared.

Figure 9 shows the achievable classification accuracy for a differentiation of sprayed vs. non-sprayed grapevine leaves measured from west and east (Figure 9a), only west side (Figure 9b) and only east side (Figure 9c) for the different times of day. Here we compare the best performing machine learning approach with the linear discrimination of the LDA. For these datasets, a machine learning approach just shows slight improvement over the linear discrimination method. Because the only condition both groups of grape plants are differing in is the spraying status with plant protection and along with the known fact of high infection pressure in this plot, detected changes in the spectral reflectance are probably determined by the counter reaction of the plants metabolism towards mildew infection. Across the results, the spectral reflectance in the VIS-NIR range seems to be the more robust predictor of the spray-status e.g., the suspected infection status. These initial results also indicate that the VIS-NIR range seems to be much less effected by time of day as well as the recording from west or east (for example SWIR at 12 p.m.). These initial results should be further investigated in the Phenoliner campaign 2017.

The correct classification rate is based on a per-pixel evaluation. For the evaluation of the whole plant, a voting of pixel results can be achieved to derive a per-plant prediction of the infection status. Furthermore, the machine learning approach will benefit from a broader database, since more confounding variations in the plant will be represented and aggregated by the machine learning model.

**Figure 10:**
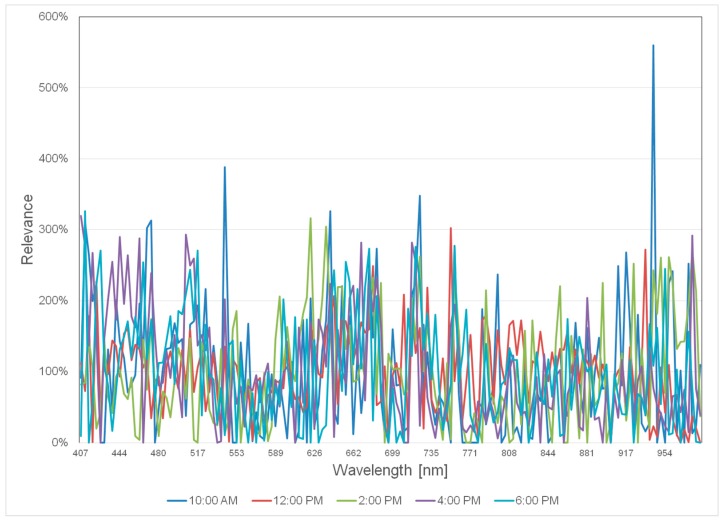
Relevance profile of the visual-near infrared range based on RBF performance at different recording times.

The machine learning approach RBF also calculates a relevance profile, which indicates what wavelength is informative for the task at hand. This technique will enable the overall goal of the project to find a set of wavelength that are informative for the pathogen detection while keeping the system flexible for new applications beyond the current scope. Figure 10 showing the relevance profile for the visual-near infrared range is depicted. The y-axis shows a percentage down- or up-regulation of wavebands according to the set tasks. As clearly visible, the profile differs greatly from an equal weighting of all wavebands. In future work we will test if this information can reduce the number of necessary wavelength.

### 3.3. Discussion of One Year Experiences with the Phenoliner

The new, robust phenotyping platform has been successfully tested in season 2016. Two sensor systems (Sensors A and B) have been implemented on the Phenoliner and the sensor data have been connected to the geo-reference to successfully link this information for phenotypic trait evaluation. The idea of a moving tunnel has been shown to work very well to overcome changing light conditions, however some small adjustments have to be added to improve the standardization of the light conditions in the tunnel. Therefore, a second curtain will be installed in the front of the machine.

#### 3.3.1. Sensor A

In season 2016 numerous rows were visually captured with the Phenoliner moving at a speed of 0.2–0.3 m/s, obtaining images with a frequency of 5 Hz. The error of image location was within a few centimetres or even below. Using this system, a near-to-complete high throughput screening of multiple vine rows is possible within one day. Future experiments need to determine the possibility to adapt the Phenoliner’s operating speed to the usual operating speed of 10–15 km/h of field working machines. The speed limit is currently restricted by the possible frequency of image acquisition and storage. To reach the required image overlap of 70–80% at a speed of 15 km/h would require an imaging frequency of 26 Hz and a data storage speed of 1.15 Gb/s for the three vertical cameras. While a realisation is generally possible, this would require high investment costs. The second restriction is eventual plant movements through enhanced airflow at greater speeds. A solution might be a pre-preparation of the vine rows but at the costs of a higher work-load.

The illumination was standardized as good as possible by closing all major openings towards the tunnel and utilizing external illumination-units. Some improvements regarding the intensity of the external illumination and the background colour still have to be carried out to enhance the quality of the point cloud. Regarding point cloud reconstruction, three phenomena are connected with a high intensity illumination. The first phenomenon is the false reconstruction of the background at the border of foreground objects. The second phenomenon is the high intensity reflection of light at the background, which may lead to oversaturation in the images. Two background colours and materials were tested during our experiments. A heavy grey tarpaulin (Figure 5b left side, black berries) and a white blanket (Figure 5b right side, green berries) constituted two alternative backgrounds. Images taken with a white background, exhibit a high level of oversaturation derived from high intensity reflections of the white background. The resulting point cloud suffers from false colours and falsely reconstructed points at object borders in the point cloud, as can be seen in Figure 5b.

**Figure 11:**
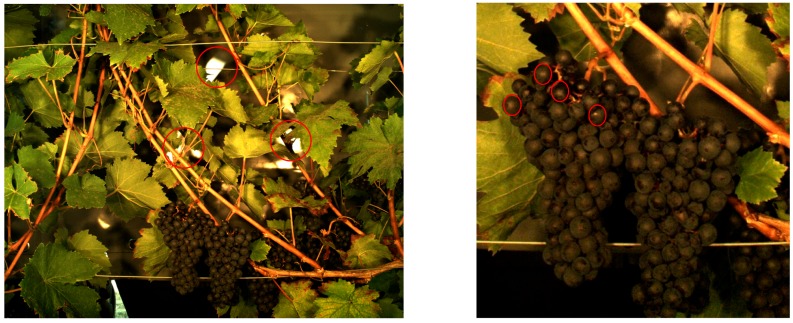
High intensity reflections result in a wrong point positions at object borders (left image). At berry arches, they may corrupt the spherical geometry of the berries during reconstruction.

In contrast, the images taken with a grey background, exhibit correct colour assignments and sharper object borders. Still, high intensity reflections at the background are visible as well and may cause background points to be reconstructed at a wrong position. The third phenomenon is the high intensity reflection at berry arches (Figure 11).

In future experiments it is planned to address these problems in two ways. First, by the utilization of polarizing filters to reduce the high intensity reflections on berry arches and background. Secondly, the background will be painted in a matt colour to further minimize reflections. For an efficient background segmentation the colour will either be pitch black or of a neon-colour shade to distinguish it from the foreground.

#### 3.3.2. Sensor B

The integration of two optical sensor systems, computer hardware and light sources in a compartment while trying not to overuse the space, but getting good results for both sensors at the same time, was one of the biggest tasks to begin with. Despite earlier observations, Sensor B had to be rotated 90° since the distance between camera (fixed focal length) and plant canopy was still too short. A mirror capable of reflecting all relevant wavelengths needed to be introduced into the optical system. After that, the system was up and running while the Phenoliner was keeping a steady velocity of 0.1 m/s. During the season, the foil covering of the reflectance pad was exposed to some stress through plant contact and will be replaced with a thin glass window. The camera acquisition data as well as the GPS data stream was generally stable not counting the several improvements that needed to be done to the acquisition software. All in all the current hardware setup delivered promising results and its use will also be extended to the laboratory. Preliminary data taken from a repeated measurement of potentially infected vines showed that a machine learning model so far does not consistently outperformed a linear discrimination of the sensor data used so far. The VIS-NIR range seems to be the better and more stable indicator for the spray-status e.g., infection status while the shortwave-infrared range was influenced by day-time and recording direction. This fact has to be evaluated in the coming campaign with plants analysed for true infection. With a growing database of hyperspectral data samples it is to expect that the machine learning approach gains robustness against confounding variations in the data. The advantage (Sensor B) of the current system is the collection of reflectance data narrowly sampled across the wavelength range from 400 to 2500 nm which sets it apart from usual multispectral approaches whose wavelengths are chosen according to spectral indices known in advance to have a correlation with the target values (for example chlorophyll, biomass, nitrogen). Machine learning is the tool to cope with the high-dimensional data produced by the system and its potential non-linear relationship with the target value.In the long run, the hyperspectral imaging system is intented to be used on a commercial platform like a tractor in a productive environment. Some prerequisites must be met before, that is a higher framerate supported by better artifical lighting allowing lower exposure times. Also, if the evidence hardens, that VIS-NIR is the spectral range holding sufficient information for the detection of grapevine diseases, this would be beneficial for the realization of a multispectral system for the commercial application. 

## 4. Conclusions

A new phenotyping platform for grapevine research has been successfully introduced. The Phenoliner was build based on a grape harvester and is equipped with two high-end visual camera systems using RGB, NIR and hyperspectral imaging to screen vines directly in the field. It has the potential to enable high-throughput phenotyping taking different phenotypic traits like yield parameters and health status into account. Compared to other phenotypic platforms it is independent of the surrounding light conditions. Furthermore the Phenoliner is very robust for field application and its functionality could be extended with additional sensors in the future.

## Figures and Tables

**Table 1:** Accuracy of spray status prediction based on a single spectrum. Machine learning models tested: Soft-max output layer (PNET), Multi-Layer Perceptron with linear output (MLP), Radial Basis Function (RBF) and network Partially Least Square (PLS) model. The best results, also shown in Figure 9, are marked in bold. Depicted are average performances on the test sets in the 5-fold cross validation.

	VNIR						SWIR			
**West and east canopy side**										
	PNET	MLP	RBF	PLS		PNET	MLP	RBF	PLS
10 a.m.	81.80%	79.40%	**86.85%**	86.55%	10 a.m.	70.95%	70.25%	77.65%	**77.80%**
12 p.m.	81.30%	81.40%	83.90%	83.05%	12 p.m.	66.65%	66.70%	72.25%	**73.95%**
2 p.m.	77.05%	77.65%	**83.40%**	80.95%	2 p.m.	60.30%	59.90%	67.95%	**69.25%**
4 p.m.	80.10%	79.75%	**84.00%**	82.45%	4 p.m.	67.30%	67.15%	**78.00%**	77.15%
6 p.m.	78.75%	78.95%	81.30%	**81.80%**	6 p.m.	63.25%	64.45%	**69.10%**	68.40%
**West side**										
	PNET	MLP	RBF	PLS		PNET	MLP	RBF	PLS
10 a.m.	81.25%	83.05%	**87.95%**	87.65%	10 a.m.	73.35%	72.05%	**84.60%**	83.95%
12 p.m.	84.90%	85.20%	**88.05%**	87.80%	12 p.m.	81.20%	80.65%	88.00%	**88.25%**
2 p.m.	79.50%	80.70%	**87.05%**	85.60%	2 p.m.	68.55%	71.45%	75.30%	**77.50%**
4 p.m.	82.95%	81.85%	**84.70%**	83.90%	4 p.m.	70.60%	73.15%	**85.60%**	84.60%
6 p.m.	83.65%	82.15%	**86.30%**	85.80%	6 p.m.	72.45%	72.20%	83.95%	**84.10%**
**East side**										
	PNET	MLP	RBF	PLS		PNET	MLP	RBF	PLS
10 a.m.	80.25%	82.05%	**85.60%**	85.15%	10 a.m.	76.20%	73.45%	**83.85%**	83.50%
12 p.m.	78.05%	79.00%	83.40%	**83.85%**	12 p.m.	64.20%	63.55%	71.75%	**73.60%**
2 p.m.	80.35%	78.55%	**84.55%**	83.45%	2 p.m.	64.90%	64.85%	70.45%	**73.90%**
4 p.m.	80.05%	79.45%	**85.55%**	83.95%	4 p.m.	71.10%	71.25%	77.95%	**80.75%**
6 p.m.	77.65%	76.70%	**80.45%**	80.15%	6 p.m.	63.55%	63.50%	68.90%	**69.50%**
